# Gd-EOB-DTPA-Enhanced MRI Combined with ALBI Score and AFP for Predicting Histologic Grade in Hepatocellular Carcinoma: A Multicentre Study from Vietnam

**DOI:** 10.3390/diagnostics16132018

**Published:** 2026-06-28

**Authors:** Van Hung Nguyen, Dang Luu Vu, The Anh Pham, Cong Long Nguyen, Van Khang Le, Ngoc Trung Nguyen, Le Minh Vu, Ham Hoi Nguyen

**Affiliations:** 1 Department of Diagnostic Imaging, Hanoi Medical University, Hanoi 100000, Vietnam; vudangluu@hmu.edu.vn (D.L.V.); drkhang2006@gmail.com (V.K.L.); 2 Department of Diagnostic Imaging, Thai Binh University of Medicine and Pharmacy, Hung Yen 17000, Vietnam; drtrung82@gmail.com; 3 Institute of Radiology and Intervention, Bach Mai Hospital, Hanoi 100000, Vietnam; 4 Department of Hepatobiliary-Pancreatic Surgery, K Hospital, Hanoi 100000, Vietnam; theanhbenhvienk@gmail.com; 5 Gastroenterology-Hepatology Center, Bach Mai Hospital, Hanoi 100000, Vietnam; nguyenconglongbvbm@gmail.com; 6 Diagnostic Imaging Center, K Hospital, Hanoi 100000, Vietnam; vuleminh156@gmail.com; 7 Digestive Surgery Center, Bach Mai Hospital, Hanoi 100000, Vietnam; hamhoint30@gmail.com

**Keywords:** hepatocellular carcinoma, Gd-EOB-DTPA, magnetic resonance imaging, histologic grade, Edmondson–Steiner, ALBI score, alpha-fetoprotein, hepatobiliary phase

## Abstract

**Objectives:** The histologic grade is an important prognostic factor in hepatocellular carcinoma (HCC). A Gd-EOB-DTPA-enhanced MRI may provide noninvasive imaging markers related to tumour differentiation. This study aimed to evaluate the association of Gd-EOB-DTPA-enhanced MRI features, together with the albumin–bilirubin (ALBI) score and alpha-fetoprotein (AFP), with the HCC histologic grade and to assess the performance of combined predictive models. **Methods:** In this prospective cross-sectional study, 75 patients (mean age, 56.4 years; 66 men) with 88 histopathologically confirmed HCC lesions were enrolled. Patients were classified into well-differentiated (grades I–II, *n* = 24) and poorly differentiated (grades III–IV, *n* = 51) groups according to the Edmondson–Steiner system. The MRIs were performed on a 1.5-T scanner and included T1-weighted in-phase/opposed-phase imaging; T2-weighted imaging; diffusion-weighted imaging; and dynamic Gd-EOB-DTPA-enhanced sequences, including arterial, portal venous, transitional, and 20 min hepatobiliary phases. Two radiologists, blinded to the pathology, assessed predefined imaging features, and the lesion-to-liver ratio (LLR) was measured. Group comparisons were performed using Student’s *t*-test, a Mann–Whitney U test, and a chi-square or Fisher’s exact test, followed by a multivariable logistic regression and ROC analysis with bootstrap resampling. **Results:** Compared with well-differentiated HCC, poorly differentiated HCC showed a higher frequency of peritumoral hepatobiliary phase (HBP) hypointensity (62.7% vs. 4.2%, *p* < 0.001) and peritumoral arterial hyperintensity (39.2% vs. 0%, *p* < 0.001). In the multivariable analysis, peritumoral HBP hypointensity remained independently associated with poorly differentiated HCC (OR = 30.89, *p* = 0.002). The two-parameter MRI model, including peritumoral HBP hypointensity and HBP tumour signal, yielded an AUC of 0.84. The combined MRI + ALBI + AFP model yielded an AUC of 0.87 and an accuracy of 78.7%, representing only a small exploratory improvement over the two-parameter MRI model (AUC = 0.84) in this cohort. **Conclusions:** Gd-EOB-DTPA-enhanced MRI features, particularly peritumoral HBP hypointensity, were associated with a high histologic grade in HCC. In this surgically treated, predominantly HBV-related cohort with mostly preserved liver function, these findings provide a preliminary basis for preoperative histologic risk stratification; however, they remain exploratory and require external validation in larger, more diverse cohorts before broader clinical application.

## 1. Introduction

Hepatocellular carcinoma (HCC) is the sixth most common cancer worldwide and the third leading cause of cancer-related deaths, with an estimated 866,000 new cases and 758,000 deaths in 2022 [1]. In Vietnam, liver cancer ranks second in incidence (24,502 cases, 13.6%) and first in cancer-related mortality (23,333 deaths, 19.4%) [1], representing a major public health burden due to the high prevalence of chronic hepatitis B virus (HBV) and hepatitis C virus (HCV) infections [2]. In this context, pre-treatment prognostic assessment depends not only on tumour size or number, but also, importantly, on tumour biology, particularly the histologic grade [3].

The Edmondson–Steiner (ES) classification is the most widely used histologic grading system for HCC [4], categorising tumours from grade I (well differentiated) to grade IV (undifferentiated). Poorly differentiated HCC (grades III–IV) has been associated with a higher rate of microvascular invasion (MVI), earlier recurrence after curative treatment, and poorer overall survival [5]. Therefore, the accurate preoperative prediction of histologic grade is of considerable clinical relevance.

Gadoxetic acid (Gd-EOB-DTPA, Primovist^®^) is a hepatocyte-specific MRI contrast agent that provides information on both dynamic contrast-enhanced phases and the hepatobiliary phase (HBP) [6]. During the HBP, Gd-EOB-DTPA is taken up through the OATP1B3 transporter, allowing for indirect assessment of hepatocellular function. Poorly differentiated HCCs exhibit reduced or absent OATP expression, resulting in characteristic hypointensity on HBP images, and the degree of signal reduction reflects the level of OATP1B3 expression in tumour cells [7]. Several studies have shown that peritumoral HBP hypointensity, peritumoral arterial hyperintensity, and LLR are associated with the histologic grade and microvascular invasion [7,8].

In addition to imaging findings, AFP has been investigated as a predictor of HCC differentiation and has been incorporated into nomograms for preoperative prediction of histologic grade [9]. AFP levels tend to increase with loss of differentiation, reflecting the biological link between AFP expression and tumour aggressiveness [10]. The ALBI score, which is derived from serum albumin and bilirubin, provides an objective assessment of hepatic reserve and has been associated with tumour behaviour through its relationship with early postoperative recurrence [11]. However, the combined value of Gd-EOB-DTPA-enhanced MRI, the ALBI score, and AFP for predicting the HCC histologic grade remains incompletely defined, and no Vietnamese study has yet evaluated predictive models for HCC histologic grading. Therefore, we conducted this study to investigate the association of Gd-EOB-DTPA MRI features, the ALBI score, and AFP with the HCC histologic grade, and to evaluate the diagnostic performance of individual parameters and combined models for predicting poorly differentiated HCC (grades III–IV).

## 2. Materials and Methods

### 2.1. Study Population

This cross-sectional analytical study was conducted at Bach Mai Hospital and K Hospital. Patients with HCC who underwent Gd-EOB-DTPA-enhanced MRI between May 2025 and January 2026 were prospectively enrolled. Eligible patients were recruited consecutively at both centres. Patients were therefore enrolled prospectively, whereas the predefined MRI features were extracted afterwards during a dedicated blinded research review of the stored images rather than from the original clinical radiology reports; the design is thus more precisely described as prospective patient recruitment with blinded research-based image review. Of the patients assessed for eligibility during the study period, those meeting the criteria below were included, yielding a final cohort of 75 patients with 88 tumours.

Inclusion criteria: (1) Histopathologically confirmed HCC based on surgical specimens; (2) Gd-EOB-DTPA-enhanced MRI performed within 4 weeks before tissue sampling; and (3) complete clinical, imaging, and laboratory data.

Exclusion criteria: (1) Prior treatment before MRI; (2) inadequate image quality; or (3) combined hepatocellular-cholangiocarcinoma. Because the study required histopathologic confirmation from surgical specimens, the cohort consisted of patients with resectable, predominantly intrahepatic disease. Patients with advanced-stage HCC (BCLC stage C) in whom extrahepatic metastases or macrovascular invasion were the dominant lesions and the intrahepatic tumour burden was minor were not surgical candidates and therefore did not meet the inclusion criteria; such cases were consequently not represented in this surgically treated population. In the small number of patients with more than one intrahepatic lesion, the largest histopathologically confirmed lesion was designated the index lesion and used for all lesion-level imaging measurements and inferential analyses.

A total of 75 patients with 88 tumours were included; however, the inferential analyses focused on the index lesion of each patient. The index lesion was defined as the largest histopathologically confirmed lesion in each patient. When more than one lesion was resected and sampled, the highest histologic grade among the sampled lesions was assigned to the patient, so that the index lesion corresponded to the lesion determining the patient’s group classification; in patients with concordant grades, the largest sampled lesion was used. Additional lesions that were not sampled histologically did not contribute to the histologic grouping and were used only for descriptive imaging analyses. This index lesion approach was adopted so that each patient contributed a single lesion to the inferential analyses, avoiding clustering of multiple lesions within the same patient.

### 2.2. Study Methods

#### 2.2.1. MRI Acquisition

The MRIs were performed on a 1.5-T scanner. Both centres used the same MRI platform (1.5-T) and an identical, harmonised acquisition protocol, including the same core sequences, the same gadoxetic acid dose and injection rate, and the same dynamic and hepatobiliary phase timing, so that the imaging parameters did not differ between centres. The imaging protocol included in-phase/opposed-phase T1-weighted imaging, T2-weighted imaging, diffusion-weighted imaging (DWI), and dynamic contrast-enhanced imaging. Gd-EOB-DTPA (gadoxetic acid disodium, Primovist^®^; Bayer Pharma AG, Berlin, Germany) was administered at a dose of 0.025 mmol/kg at a rate of 1 mL/s, followed by a 20 mL saline flush. The imaging phases included the arterial phase (20–35 s), portal venous phase (60–80 s), transitional phase (3 min), and hepatobiliary phase (20 min) [12].

#### 2.2.2. Image Analysis

**Figure 1:**
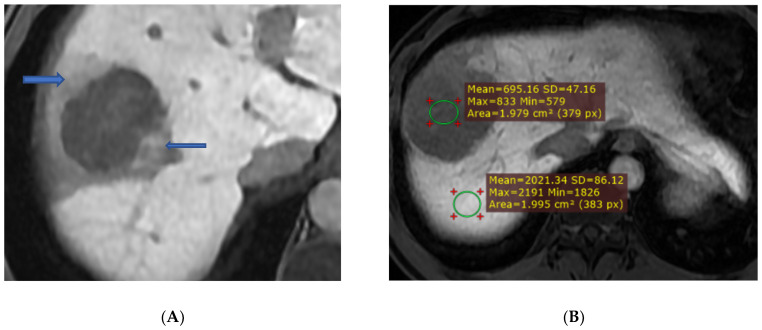
Axial hepatobiliary-phase (20 min) gadoxetic acid (Gd-EOB-DTPA)-enhanced T1-weighted images in two different patients, both with Edmondson–Steiner grade III HCC, illustrating peritumoral HBP hypointensity and the region-of-interest (ROI)-based measurement of the lesion-to-liver ratio (LLR). The LLR is defined as the ratio of the mean signal intensity within the tumour ROI to that within an ROI placed in the adjacent liver parenchyma on the same slice. (**A**) The signal-reduction margins around the tumour (HBP), corresponding to the peritumoral HBP hypointensity, are areas of decreased signal compared to healthy liver parenchyma, but still show an increased signal compared to the tumour (blue arrows). (**B**) Illustration of measurement of the signal ROI of the tumour (excluding necrotic and bleeding areas of the tumour) and healthy liver parenchyma on the same slice. In panel B, the green circular regions indicate the tumour and adjacent liver parenchymal ROIs used to calculate the LLR.

Two radiologists with more than 5 years of experience independently reviewed all the MRI studies while blinded to the histopathologic results. The following imaging features were assessed: APHE, washout, and capsule appearance according to LI-RADS 2018 criteria [13]; peritumoral arterial hyperintensity; peritumoral HBP hypointensity; tumour signal intensity during HBP; and the lesion-to-liver ratio (LLR), defined as the ratio of tumour signal intensity to adjacent liver parenchymal signal intensity on HBP images. Because HCC, particularly poorly differentiated tumours, frequently shows marked intratumoral heterogeneity during the HBP (Figure 1), ROI placement was standardised to minimise measurement variability. For each lesion, a single round or oval ROI of at least 1.5 cm^2^ was placed on the section showing the largest tumour cross-section, positioned within the solid tumour component while deliberately excluding cystic, necrotic, haemorrhagic, and obviously heterogeneous areas as well as the peritumoral signal-reduction margin. To account for heterogeneity, two senior radiologists reviewed all sequences side by side and reached the final ROI position by consensus, and a corresponding reference ROI of comparable size was placed in the adjacent liver parenchyma on the same slice, avoiding large vessels, bile ducts, and artefacts. Care was taken to keep the tumour ROI representative of the dominant tumour signal rather than sampling only the most hypointense focus, so that the LLR reflected the overall lesion rather than a single extreme region. The two radiologists assessed all images independently while blinded to the histopathologic and clinical data. The interobserver agreement for the categorical imaging features, including the key predictor of peritumoral HBP hypointensity, was good (Cohen’s κ = 0.86), and any disagreements were resolved by consensus, with the consensus reading used in all subsequent analyses. Representative hepatobiliary phase MRI findings and the region-of-interest (ROI)-based approach used for the lesion-to-liver ratio (LLR) measurement are shown in Figure 1.

#### 2.2.3. Histopathology

Histopathologic grading was performed according to the Edmondson–Steiner classification. For heterogeneous tumours, the highest histologic grade was recorded. The tumours were then categorised into two groups: well-differentiated (grades I–II) and poorly differentiated (grades III–IV).

#### 2.2.4. Clinical and Biochemical Variables

The clinical and laboratory variables included age, sex, HBV/HCV status, cirrhosis, AFP level (ng/mL), bilirubin (µmol/L), albumin (g/L), AST, and ALT (U/L). The ALBI score was calculated as ALBI = (log10 bilirubin × 0.66) + (albumin × −0.085) [11]. For the categorical analyses, serum AFP was dichotomised at 20 ng/mL. This threshold was prespecified as a conventional, clinically interpretable cut-off frequently used in HCC studies and was not selected by ROC optimisation in the present cohort [14].

The ALBI grades were defined as follows: grade 1 (≤−2.60), grade 2 (>−2.60 to ≤−1.39), and grade 3 (>−1.39).

#### 2.2.5. Pathologic Assessment

**Figure 2:**
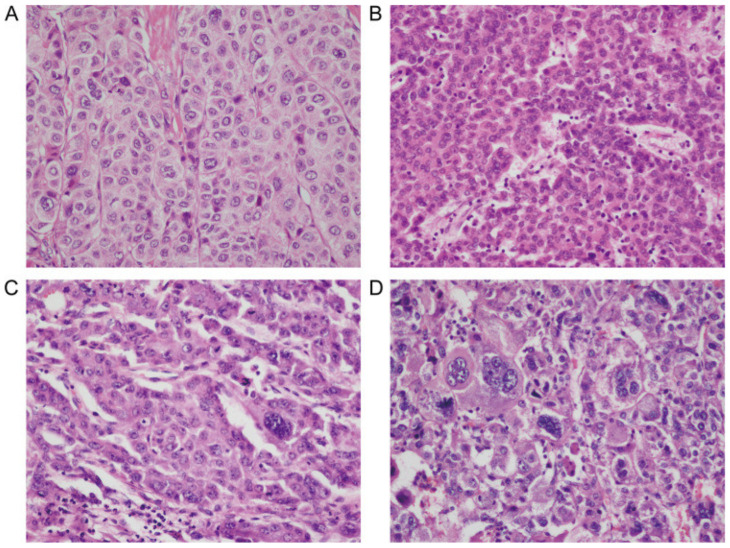
Representative histopathologic appearances of hepatocellular carcinomas across the Edmondson–Steiner spectrum. (**A**) Well-differentiated tumour with relatively preserved trabecular architecture and mild nuclear atypia; (**B**) moderately differentiated tumour with increased cellularity and nuclear irregularity; (**C**) poorly differentiated tumour with marked architectural distortion; (**D**) high-grade tumour with pronounced pleomorphism and marked atypia. Panels correspond to Edmondson–Steiner grades I (**A**), II (**B**), III (**C**), and IV (**D**). Haematoxylin and eosin staining; original magnification ×200.

The histopathologic findings were evaluated by a gastrointestinal/hepatobiliary pathologist with more than 5 years of experience, independently from and blinded to the MRI findings. To improve the reproducibility of grading, all specimens underwent a second independent review by another gastrointestinal/hepatobiliary pathologist, and the Edmondson–Steiner grade and microvascular invasion status were confirmed; discordant cases were re-examined jointly and resolved by consensus. Additional recorded features included true microvascular invasion (MVI), microscopic tumour size, and parenchymal fibrosis. Representative histopathologic appearances across the Edmondson–Steiner spectrum are shown in Figure 2.

### 2.3. Statistical Analysis

The normally distributed continuous variables were expressed as the mean ± standard deviation and compared using a Student’s *t* test. The non-normally distributed variables were expressed as the median (interquartile range) and compared using a Mann–Whitney U test. The normality of continuous variables was assessed using a Shapiro–Wilk test together with inspection of histograms; on this basis, the tumour size, LLR, ALBI score, albumin, bilirubin, and AFP were treated as non-normally distributed. Because AFP was markedly right skewed, it was additionally examined after a natural-log transformation. The categorical variables were expressed as frequency (%) and compared using a chi-square test or Fisher’s exact test. The variables with *p* < 0.10 on a univariable analysis were entered into a multivariable logistic regression. Given the limited number of events, a parsimonious model was prespecified and candidate predictors were entered simultaneously rather than by stepwise selection; the continuous predictors were kept at their original scale, and ordinal imaging scores were entered as ordinal variables. Multicollinearity among the predictors was evaluated using pairwise correlations and variance inflation factors (VIFs), all of which were below 2, indicating no serious collinearity. An ROC analysis was performed to calculate the AUC, sensitivity, specificity, positive predictive value (PPV), negative predictive value (NPV), and accuracy. Ninety-five percent confidence intervals (CIs) for the AUCs were obtained by bootstrap resampling (2000 resamples), and the difference in the AUC between the 2-parameter and 4-parameter models was assessed using the same bootstrap procedure. Model calibration was evaluated using a Hosmer–Lemeshow goodness-of-fit test. Because of the limited sample size, the reported performance metrics should be regarded as apparent (internal) estimates that may be optimistic in the absence of external validation. A *p*-value < 0.05 was considered statistically significant. Statistical analyses were conducted using SPSS version 27.0.

### 2.4. Ethical Considerations

This study was conducted in accordance with the Declaration of Helsinki and was approved by the Biomedical Ethics Committee of Bach Mai Hospital (IRB00009695). Patients and their families were thoroughly informed about the study objectives and procedures. Participants were free to withdraw from the study at any time.

## 3. Results

The clinical and biochemical characteristics of the study cohort are summarized in Table 1. A total of 75 patients with HCC were included: 24 (32.0%) with well-differentiated tumours (grades I–II) and 51 (68.0%) with poorly differentiated tumours (grades III–IV).

The patients with poorly differentiated HCC according to the Edmondson–Steiner classification (ES III–IV) had significantly lower serum albumin levels than those with ES I–II tumours (41.72 ± 3.06 vs. 43.92 ± 2.57 g/L, *p* = 0.002). The proportion of patients with ALBI grades 2–3 was also higher in the ES III–IV group, whereas age, sex, HBV/HCV status, cirrhosis, AFP, bilirubin, and the continuous ALBI score did not differ significantly between groups.

Baseline liver function was well preserved and similar between groups. Almost all patients were Child–Pugh class A (74/75), with only one patient (in the poorly differentiated group) classified as class B (*p* = 1.00), and the MELD score did not differ significantly between groups (median 6 [IQR 6–7] vs. 7 [IQR 6–8], *p* = 0.07). These findings indicate that the present cohort consisted predominantly of patients with well-compensated, surgically treated HCC, which should be considered when interpreting the ALBI- and signal-based imaging metrics described below.

The Gd-EOB-DTPA MRI features according to Edmondson–Steiner group are summarized in Table 2.

The ES III–IV group showed a more aggressive imaging profile on the Gd-EOB-DTPA-enhanced imaging, including larger tumour size, more frequent peritumoral arterial hyperintensity, more frequent peritumoral HBP hypointensity, a higher proportion of HBP hypointensity, and lower HBP LLR values. In contrast, the tumour number, APHE, capsule appearance, and washout did not differ substantially between the two groups.

The univariable logistic regression results for predicting high histologic grade HCC are presented in Table 3.

In the univariable analysis, the peritumoral HBP hypointensity, HBP tumour signal intensity, LLR, and elevated AFP (≥20 ng/mL) were significantly associated with high histologic grade HCC (ES III–IV). Among these, the peritumoral HBP hypointensity showed the strongest association (OR = 38.74, *p* < 0.001). In contrast, the ALBI score, tumour size, and APHE did not show significant associations in the present dataset.

The multivariable logistic regression results for predicting high histologic grade HCC are shown in Table 4.

A multivariable regression was performed on the index lesion of 75 patients; the entire cohort comprised 88 tumours on MRI. In the multivariable model, the peritumoral HBP hypointensity remained an independent predictor of high histologic grade HCC (ES III–IV) (OR = 30.89; 95% CI: 3.54–269.66; *p* = 0.002). The HBP tumour signal intensity showed a trend in the same direction but did not reach statistical significance (*p* = 0.10), while elevated AFP and ALBI scores were not independently associated.

The preliminary total score formula is as follows:

Total score = 100 × (peritumoral HBP hypointensity) + 21 × (HBP tumour signal level) + 17 × (AFP < 20 ng/mL) + 30 × (ALBI + 3.5)

where the peritumoral HBP hypointensity = 1 if present, 0 if absent; the HBP tumour signal level = 0, 1, or 2; and AFP < 20 = 1 if AFP < 20 ng/mL, 0 if AFP ≥ 20 ng/mL. This total score formula is presented only as an exploratory illustration of how the retained variables might be combined; the weights were derived directly from the regression coefficients in the present small cohort and have not been internally or externally validated, and no operating cut-off was established. It should therefore be regarded as hypothesis generating rather than as a ready-to-use clinical score.

The diagnostic performance of the individual parameters and combined predictive models is summarized in Table 5.

Among the individual parameters, the peritumoral HBP hypointensity showed the highest diagnostic performance (AUC = 0.79; 95% CI: 0.72–0.87), followed by the HBP tumour signal intensity (AUC = 0.72; 95% CI: 0.59–0.82); the tumour size (0.67), elevated AFP (0.57) and ALBI grades 2–3 (0.58) were weaker. Combining the two imaging markers yielded a two-parameter MRI model with an AUC of 0.84 (95% CI: 0.74–0.91). The addition of elevated AFP and ALBI grades 2–3 was associated with a numerically higher AUC of 0.87 (95% CI: 0.78–0.94); however, the gain over the two-parameter MRI model was small and not statistically robust (ΔAUC = 0.035; 95% bootstrap CI: −0.004 to 0.080). Both models showed adequate calibration (Hosmer–Lemeshow *p* = 0.22 and *p* = 0.41 for the two- and four-parameter models, respectively). These results indicate that the imaging-based model carried most of the discriminative information, and that the apparent incremental value of AFP and ALBI should be interpreted with caution given the small, imbalanced sample and the very small number of ALBI grades 2–3 cases.

## 4. Discussion

Our results suggest that hepatobiliary phase imaging features, particularly peritumoral HBP hypointensity and reduced tumour signal on HBP images, are associated with poorly differentiated HCC.

A notable finding of this study is the association between peritumoral HBP hypointensity and poorly differentiated HCC, with an estimated OR of 38.74. Peritumoral HBP hypointensity has been regarded as an imaging marker of microvascular invasion, reflecting the invasive biological behaviour of a tumour. The proposed mechanism is that microscopic tumour thrombi in the peritumoral portal venules reduce or obstruct portal venous flow, leading to impaired hepatocyte function and decreased uptake of Gd-EOB-DTPA in the surrounding liver tissue, thereby creating a hypointense rim relative to the adjacent liver parenchyma. The high specificity (95.8%) and PPV (97.0%) of this sign suggest that it may reflect a more aggressive tumour biology, in keeping with previous reports [15,16].

The degree of hepatobiliary phase tumour hypointensity was another imaging feature associated with the histologic grade (OR = 3.16, *p* = 0.002), and its quantitative counterpart, LLR, also showed predictive value, possibly reflecting a progressive loss of OATP1B3 expression during dedifferentiation. As HCC progresses from well-differentiated to poorly differentiated forms, the expression of hepatocyte-specific membrane transporters gradually decreases, leading to the reduced uptake of Gd-EOB-DTPA [7]. The lower LLR observed in poorly differentiated HCC (0.47 vs. 0.62) may therefore reflect this progressive loss of OATP1B3 expression during tumour dedifferentiation. The LLR also offers the advantage of being an objective measurement that is less dependent on scanner-specific signal intensity, as it represents a relative ratio between the tumour and background liver signal on the same image slice.

Peritumoral arterial hyperintensity was observed only in the poorly differentiated group (39.2% vs. 0%, *p* < 0.001) in the univariable analysis but did not remain significant in the multivariable model (*p* = 0.42), possibly because of collinearity with peritumoral HBP hypointensity, as both features have been linked to microvascular invasion [15]. The poorly differentiated HCC also had a larger tumour size (*p* = 0.02), although tumour size was not independently associated in the multivariable model, suggesting that it may function more as a surrogate marker than as a direct indicator of tumour aggressiveness. Washout on the portal venous phase showed a non-significant trend toward being more frequent in poorly differentiated tumours (*p* = 0.06), consistent with decreased portal venous supply and increased arterial perfusion in more aggressive lesions [12].

The ALBI score is an objective model based on two routine blood biomarkers, albumin and bilirubin. Although it was not independently associated with the histologic grade in the multivariable model, lower albumin levels were observed in the poorly differentiated group (*p* = 0.002), which may indicate a relationship between hepatic functional reserve and tumour behaviour. A plausible explanation is that the lower albumin and slightly higher ALBI values observed in the poorly differentiated group reflect the background liver condition rather than a direct biological effect of tumour grade. Poorly differentiated HCC tends to arise from a background of more advanced chronic liver disease, in which reduced hepatic synthetic capacity lowers serum albumin; larger and more aggressive tumours may also contribute through a greater systemic inflammatory and catabolic burden. The albumin difference between the groups was modest (43.9 vs. 41.7 g/L), and both groups were predominantly ALBI grade 1, so this association should be interpreted with caution. Importantly, because the two groups were not matched for tumour size or for baseline liver function, the apparent relationship between a higher ALBI score and poorly differentiated HCC may have been confounded by differences in background liver condition; for example, the poorly differentiated group also had significantly larger tumours. We were unable to fully adjust for these factors given the limited sample size, and analyses stratified or matched by tumour size or by Child–Pugh class were not feasible in the present cohort. The relationship between a higher ALBI score and recurrence after surgery was also reported by Demirtas et al. [11], who identified ALBI as a predictor of survival, recurrence, and post-hepatectomy liver failure. Zhou et al. (2023) [9] also incorporated ALBI into a nomogram to predict the histologic grade, using it as an objective alternative to the Child–Pugh classification for assessing liver functional reserve. Overall, ALBI is currently used mainly as a marker of liver function and prognosis.

In the present study, there was a trend towards a higher AFP in the poorly differentiated group (median 42.4 vs. 9.2 ng/mL), but this difference did not reach statistical significance in the between-group comparison (*p* = 0.10), likely reflecting the small, imbalanced sample and the markedly skewed AFP distribution; the analysis on a natural-log scale did not change this conclusion (*p* = 0.17). When dichotomised at 20 ng/mL, an elevated AFP was associated with a high histologic grade in the univariable regression (OR = 2.80, *p* = 0.04), although its discriminative ability was modest (AUC = 0.57) and it was not independently associated with the grade in the multivariable model. The 20 ng/mL threshold was selected a priori as a conventional clinical and prognostic cut-off rather than being optimised against the histologic grade in this cohort [14]. This prespecified approach preserved clinical interpretability and limited data-driven overfitting; nevertheless, dichotomisation may discard information, which is why AFP was also analysed as a continuous variable and after a natural-log transformation. Previous studies have also reported an association between AFP level and histologic grade in HCC. In a large-scale study of 78,743 HCC patients from the SEER database, Bai et al. [17] showed that a positive AFP at diagnosis was an independent risk factor for higher histologic grade (OR = 2.559; 95% CI: 2.075–3.157; *p* < 0.001). This study also demonstrated that AFP was an independent predictor of mortality in both the surgical and non-surgical groups. Similarly, Edoo et al. [18] reported that mean AFP levels were highest in poorly differentiated HCC, confirming a positive relationship between AFP level and degree of dedifferentiation.

Recently, several preoperative prediction models for the HCC histologic grade have been published using combined imaging and biomarker data. Zhou et al. [9] developed a nomogram for predicting ES III–IV in 240 HCC patients, integrating AFP, des-γ-carboxy prothrombin, HBsAg, HCV antibody, ALRI, and macrovascular invasion on imaging. In that study, AFP and DCP were identified as independent predictors of ES III–IV, and the proportion of patients with both AFP and DCP positivity was significantly higher in the MVI and ES III–IV groups. Compared with the model reported by Zhou et al., our study additionally evaluated hepatobiliary phase-specific imaging features, including peritumoral HBP hypointensity and reduced HBP tumour signal, which are not available on CT or extracellular contrast-enhanced MRI [12].

Other serum biomarkers may complement the total AFP in the assessment of HCC biology. Des-γ-carboxy prothrombin (DCP), also known as a protein induced by vitamin K absence or antagonist-II (PIVKA-II), has been associated with aggressive tumour behaviour and vascular invasion; notably, DCP and AFP were both retained as predictors of ES III–IV in the nomogram reported by Zhou et al. [9] AFP-L3, the Lens culinaris agglutinin-reactive fraction of AFP, has been associated with an advanced tumour phenotype and portal vein invasion [19], and the AFP-L3 percentage and absolute AFP-L3 concentration have shown stronger correlations with the HCC differentiation grade than the total AFP in some cohorts [20]. DCP/PIVKA-II and AFP-L3 were not measured consistently at both participating centres and therefore could not be evaluated reliably without introducing substantial missing data and selection bias. Future prospective multicentre studies should standardise their measurements and determine whether they provide incremental discrimination, calibration, and clinical utility beyond the Gd-EOB-DTPA-enhanced MRI features, total AFP, and ALBI.

In the field of radiomics, Yan et al. [21] developed a multiphase Gd-EOB-DTPA-enhanced MRI radiomics signature to predict the HCC histologic grade in 405 patients, with AFP as an important input variable alongside the radiomic features. Similarly, Mao et al. (2022) [22] constructed an artificial neural network (ANN) model combining the radiomic features of Gd-EOB-DTPA radiometric imaging in the hepatobiliary phase with AFP, achieving an AUC of 0.941 for differentiating high and low histological grade HCC. In that study, the AFP-integrated ANN-HBP model outperformed the logistic regression model (AUC 0.941 vs. 0.819; *p* = 0.001), suggesting that AFP may add discriminative value when combined with hepatobiliary phase imaging features. This is broadly consistent with the present findings, in which AFP alone showed modest discriminative ability, whereas the combined model yielded an AUC of 0.87 and an accuracy of 78.7%. He et al. [23] used Gd-EOB-DTPA-enhanced T1 mapping combined with ADC values from DWI. They found that ΔT1 decreased progressively as histologic differentiation worsened, and the combined model achieved an AUC of 0.811. Overall, the AUC range of 0.84–0.87 in our study is comparable to that reported in recent studies and suggests that combining conventional imaging features with AFP and ALBI may be feasible in settings where advanced radiomics infrastructure is not routinely available.

Although ALBI was not independently associated with the histologic grade in the multivariable analysis, inclusion of ALBI together with peritumoral HBP hypointensity, tumour HBP signal, and AFP produced a combined model with an AUC of 0.87, a specificity of 87.5%, and an accuracy of 78.7%, which was higher than that of the two-parameter MRI model alone (AUC 0.84). This suggests that combining imaging features with laboratory variables may improve model performance. This is particularly relevant in routine clinical practice because ALBI is derived from albumin and total bilirubin, which are inexpensive, widely available laboratory tests, and AFP is routinely performed. Accordingly, this integrated model may be relevant to preoperative assessment workflows, particularly in settings with variable resource availability; however, further validation is needed. The combined models showed better discriminative performance than the individual parameters in this dataset. The two-parameter MRI model achieved an AUC of 0.84 and a specificity of 95.8%. The integrated MRI + ALBI + AFP model yielded an AUC of 0.87 and an accuracy of 78.7%, suggesting that adding simple laboratory variables to a focused MRI model may improve discrimination. Preoperative identification of high histologic grade HCC may support preoperative risk stratification and may be relevant to treatment planning and follow-up strategies [24,25].

This study has several limitations. First, the sample size was relatively small (*n* = 75), leading to a wide confidence interval for the OR of peritumoral HBP hypointensity (4.83–310.40). Second, the distribution between groups was unbalanced, and the study population consisted predominantly of surgically treated HBV-related HCC. Third, the two groups were not matched for tumour size or for background liver function, and the poorly differentiated group had both larger tumours and lower serum albumin; consequently, the observed associations of ALBI and albumin with histologic grade may be partly confounded by the underlying liver condition, and they should be regarded as exploratory rather than causal. Future studies with larger samples should adjust for, stratify by, or match on tumour size and baseline liver function (for example, Child–Pugh class) to better isolate the relationship between these markers and tumour grade. Fourth, the cohort was almost exclusively Child–Pugh class A with few cirrhotic patients, so the extent to which impaired liver function modifies signal-based metrics, such as LLR and HBP tumour signal—which are measured relative to the background liver—could not be adequately evaluated; the models therefore apply mainly to well-compensated, surgically treated HBV-related HCC, and residual confounding and effect modification by liver function and cirrhosis cannot be excluded. Fifth, although ROI placement was standardised and determined by consensus, ROI-based measurement in heterogeneous HCC lesions may remain partly operator dependent, and future studies should assess the reproducibility of quantitative LLR measurements. Sixth, although interobserver agreement for the key imaging features was good (κ = 0.86) and the models showed acceptable apparent calibration, the reported discrimination and calibration are internal estimates that were not corrected for optimism and were not externally validated; given the small, imbalanced sample, the incremental value of AFP and ALBI over the imaging-only model, in particular, requires confirmation. Seventh, DCP/PIVKA-II and AFP-L3 were not measured consistently across the two participating centres and were therefore not included in the analyses; their absence may have limited the biomarker component of the combined model. The proposed total score formula is exploratory and is not intended for clinical use in its current form. Larger prospective multicentre studies with external validation are needed to confirm these findings and to better define their clinical applicability.

## 5. Conclusions

In this two-centre cohort of surgically treated, predominantly HBV-related HCC with mostly preserved (Child–Pugh A) liver function, Gd-EOB-DTPA-enhanced MRI features—particularly peritumoral hepatobiliary phase hypointensity and reduced tumour signal on HBP images—were associated with a high histologic grade, and peritumoral HBP hypointensity showed the largest estimated independent association in the multivariable model. The combined models showed moderate-to-good apparent discrimination (AUC 0.84–0.87) with acceptable calibration, although the incremental contribution of AFP and ALBI over the imaging-only model was small and not statistically robust. These findings provide a preliminary, hypothesis-generating basis for preoperative histologic risk stratification in this specific setting and might, in principle, inform decisions such as resection strategy, consideration of neoadjuvant or adjuvant approaches, and the intensity of postoperative surveillance. However, the evidence remains exploratory, and external validation, prospective assessment of interobserver reproducibility, and formal calibration in more diverse populations are required before any broader clinical use.

## Figures and Tables

**Table 1:** Clinical and biochemical characteristics according to ES group (*n* = 75).

Variable	Grades I–II (*n* = 24)	Grades III–IV (*n* = 51)	*p*-Value
Demographic characteristics, mean ± SD			
Age (years)	54.46 ± 12.65	57.35 ± 10.64	0.39
Sex, *n* (%)			
Male	22 (91.7)	44 (86.3)	0.77
Female	2 (8.3)	7 (13.7)	0.77
Medical history and risk factors, *n* (%)			
Hepatitis C	3 (12.5)	7 (13.7)	0.90
Hepatitis B	20 (83.3)	40 (78.4)	0.76
Underlying cirrhosis	8 (33.3)	13 (25.5)	0.58
Biochemical markers, mean ± SD or median (IQR)			
AFP (ng/mL)	9.2 (4.6–110.2)	42.4 (10.1–361.2)	0.10
Bilirubin (µmol/L)	13.02 ± 4.75	11.90 ± 5.87	0.28
Albumin (g/L)	43.92 ± 2.57	41.72 ± 3.06	0.002
Liver functional reserve			
ALBI score, mean ± SD	−3.01 ± 0.20	−2.90 ± 0.36	0.11
ALBI grade, *n* (%)			
Grade 1	24 (100)	43 (84.3)	0.05
Grades 2–3	0 (0.0)	8 (15.7)	
Child–Pugh class, *n* (%)			
Class A	24 (100)	50 (98.0)	1.00
Class B	0 (0.0)	1 (2.0)	
MELD score, median (IQR)	6 (6–7)	7 (6–8)	0.07

SD: standard deviation; IQR: interquartile range. MELD: Model for End-Stage Liver Disease. Child–Pugh class and MELD score reflect baseline liver function; the cohort was predominantly Child–Pugh class A, indicating well-compensated liver disease.

**Table 2:** Gd-EOB-DTPA MRI features according to ES group (*n* = 75).

Imaging Feature	Grades I–II (*n* = 24)	Grades III–IV (*n* = 51)	*p*-Value
Tumour size			
Largest tumour diameter (mm), mean ± SD	45.88 ± 26.02	55.02 ± 24.27	0.02
Number of tumours per patient	27	61	
Tumour number, *n* (%)			
1 lesion	21 (87.5)	43 (84.3)	0.72
≥2 lesions	3 (12.5)	8 (15.7)	
APHE, *n* (%)			
Present	23 (95.8)	46 (90.2)	0.66
Absent	1 (4.2)	5 (9.8)	
Washout, *n* (%)			
Present	16 (66.7)	45 (88.2)	0.06
Absent	8 (33.3)	6 (11.8)	
Capsule appearance, *n* (%)			
Present	14 (58.3)	30 (58.8)	0.97
Absent	10 (41.7)	21 (41.2)	
Hepatobiliary phase tumour signal, *n* (%)			
Hypointense	7 (29.2)	36 (70.6)	0.002
Hyperintense	11 (45.8)	11 (21.6)	
Isointense/non-specific	6 (25.0)	4 (7.8)	
Tumour-to-liver signal ratio (LLR), mean ± SD	0.62 ± 0.29	0.47 ± 0.13	0.03
Peritumoral arterial hyperintensity, *n* (%)			
Present	0 (0.0%)	20 (39.2%)	<0.001
Absent	24 (100%)	31 (60.8%)	
Peritumoral HBP hypointensity, *n* (%)			
Present	1 (4.2%)	32 (62.7%)	<0.001
Absent	23 (95.8%)	19 (37.3%)	
Quantitative index			
HBP LLR, mean ± SD	0.62 ± 0.29	0.47 ± 0.13	0.03

APHE: arterial phase hyperenhancement; HBP: hepatobiliary phase; LLR: lesion-to-liver ratio. The entire cohort comprised 88 tumours on MRI; however, the comparisons in this table are performed at the patient/index-lesion level.

**Table 3:** Univariable logistic regression analysis for predicting high histologic grade HCC (ES III–IV).

Predictor	B Coefficient	SE	OR	95% CI	*p*-Value
Peritumoral HBP hypointensity					
No			1.00	Reference	<0.001
Yes	3.66	1.06	38.74	4.83–310.39	
HBP tumour signal intensity	1.15	0.37	3.16	1.52–6.54	0.002
Tumour size	0.18	0.12	1.19	0.94–1.52	0.149
HBP LLR	0.38	0.15	1.46	1.09–1.95	0.01
Elevated AFP (≥20 ng/mL)					
No			1.00	Reference	0.04
Yes	1.03	0.51	2.80	1.03–7.60	
Liver function (ALBI)					
ALBI score	1.20	0.85	3.32	0.62–17.71	0.149

**Table 4:** Multivariable logistic regression analysis for predicting high histologic grade HCC (ES III–IV).

Predictor	B Coefficient	SE	OR	95% CI	*p*-Value
Peritumoral HBP hypointensity					
No			1.00	Reference	0.002
Yes	3.43	1.11	30.89	3.54–269.66	
HBP tumour signal intensity	0.72	0.44	2.06	0.87–4.87	0.10
Elevated AFP (≥20 ng/mL)					
No			1.00	Reference	0.38
Yes	−0.58	0.66	0.56	0.15–2.04	
Liver function (ALBI)					
ALBI score	1.03	1.01	2.81	0.39–20.32	0.31
Constant	2.36	3.03			0.44

**Table 5:** Diagnostic performance of individual parameters and combined models for predicting poorly differentiated HCC.

Predictive Model	AUC	Se (%)	Sp (%)	PPV (%)	NPV (%)	Accuracy (%)
Individual parameters						
Tumour size (cm)	0.67	90.2	45.8	78.0	68.8	76.0
APHE	0.53	9.8	95.8	83.3	33.3	37.3
Peritumoral HBP hypointensity	0.79	62.7	95.8	97.0	54.8	73.3
HBP tumour signal intensity	0.72	70.6	70.8	83.7	53.1	70.7
Elevated AFP (≥20 ng/mL)	0.57	76.5	37.5	72.2	42.9	64.0
ALBI grades 2–3	0.58	15.7	100	100	35.8	42.7
Combined models						
2-parameter MRI model	0.84	62.7	95.8	97.0	54.8	73.3
4-parameter combined model	0.87	74.5	87.5	92.7	61.8	78.7

AUC: area under the ROC curve; Se: sensitivity; Sp: specificity; PPV: positive predictive value; NPV: negative predictive value; 2-parameter MRI model: peritumoral HBP hypointensity + HBP tumour signal intensity; 4-parameter combined model: peritumoral HBP hypointensity + HBP tumour signal intensity + elevated AFP + ALBI grades 2–3. Bootstrap 95% CIs for the AUCs (2000 resamples): peritumoral HBP hypointensity, 0.72–0.87; HBP tumour signal, 0.59–0.82; tumour size, 0.52–0.80; elevated AFP, 0.46–0.69; ALBI grades 2–3, 0.53–0.63; 2-parameter model, 0.74–0.91; 4-parameter model, 0.78–0.94. Performance metrics are apparent (internal) estimates without external validation.

## Data Availability

The data presented in this study are available from the corresponding author on reasonable request. The data are not publicly available because they contain potentially identifiable clinical information.

## References

[1] Bray F., Laversanne M., Sung H., Ferlay J., Siegel R.L., Soerjomataram I., Jemal A. (2024). Global cancer statistics 2022: GLOBOCAN estimates of incidence and mortality worldwide for 36 cancers in 185 countries. CA A Cancer J. Clin..

[2] Huy Do S. (2015). Epidemiology of Hepatitis B and C Virus Infections and Liver Cancer in Vietnam. Euroasian J. Hepato-Gastroenterol..

[3] Martins-Filho S.N., Paiva C., Azevedo R.S., Alves V.A.F. (2017). Histological Grading of Hepatocellular Carcinoma-A Systematic Review of Literature. Front. Med..

[4] Edmondson H.A., Steiner P.E. (1954). Primary carcinoma of the liver: A study of 100 cases among 48,900 necropsies. Cancer.

[5] Rodríguez-Perálvarez M., Luong T.V., Andreana L., Meyer T., Dhillon A.P., Burroughs A.K. (2013). A systematic review of microvascular invasion in hepatocellular carcinoma: Diagnostic and prognostic variability. Ann. Surg. Oncol..

[6] Van Beers B.E., Pastor C.M., Hussain H.K. (2012). Primovist, Eovist: What to expect?. J. Hepatol..

[7] Kitao A., Matsui O., Yoneda N., Kozaka K., Shinmura R., Koda W., Kobayashi S., Gabata T., Zen Y., Yamashita T. (2011). The uptake transporter OATP8 expression decreases during multistep hepatocarcinogenesis: Correlation with gadoxetic acid enhanced MR imaging. Eur. Radiol..

[8] Lee S., Kim S.H., Lee J.E., Sinn D.H., Park C.K. (2017). Preoperative gadoxetic acid-enhanced MRI for predicting microvascular invasion in patients with single hepatocellular carcinoma. J. Hepatol..

[9] Zhou Z., Cao S., Chen C., Chen J., Xu X., Liu Y., Liu Q., Wang K., Han B., Yin Y. (2023). A Novel Nomogram for the Preoperative Prediction of Edmondson-Steiner Grade III-IV in Hepatocellular Carcinoma Patients. J. Hepatocell. Carcinoma.

[10] Samban S.S., Hari A., Nair B., Kumar A.R., Meyer B.S., Valsan A., Vijayakurup V., Nath L.R. (2024). An Insight Into the Role of Alpha-Fetoprotein (AFP) in the Development and Progression of Hepatocellular Carcinoma. Mol. Biotechnol..

[11] Demirtas C.O., D’Alessio A., Rimassa L., Sharma R., Pinato D.J. (2021). ALBI grade: Evidence for an improved model for liver functional estimation in patients with hepatocellular carcinoma. JHEP Rep. Innov. Hepatol..

[12] Choi J.Y., Lee J.M., Sirlin C.B. (2014). CT and MR imaging diagnosis and staging of hepatocellular carcinoma: Part II. Extracellular agents, hepatobiliary agents, and ancillary imaging features. Radiology.

[13] Chernyak V., Fowler K.J., Kamaya A., Kielar A.Z., Elsayes K.M., Bashir M.R., Kono Y., Do R.K., Mitchell D.G., Singal A.G. (2018). Liver Imaging Reporting and Data System (LI-RADS) Version 2018: Imaging of Hepatocellular Carcinoma in At-Risk Patients. Radiology.

[14] Hsu C.-Y., Liu P.-H., Lee Y.-H., Hsia C.-Y., Huang Y.-H., Lin H.-C., Chiou Y.-Y., Lee F.-Y., Huo T.-I. (2015). Using Serum α-Fetoprotein for Prognostic Prediction in Patients with Hepatocellular Carcinoma: What Is the Most Optimal Cutoff?. PLoS ONE.

[15] Ahn S.Y., Lee J.M., Joo I., Lee E.S., Lee S.J., Cheon G.J., Han J.K., Choi B.I. (2015). Prediction of microvascular invasion of hepatocellular carcinoma using gadoxetic acid-enhanced MR and ^18^F-FDG PET/CT. Abdom. Imaging.

[16] Kim K.A., Kim M., Jeon H.M., Kim K.S., Choi J., Ahn S.H., Cha S.J., Chung Y.E. (2012). Prediction of microvascular invasion of hepatocellular carcinoma: Usefulness of peritumoral hypointensity seen on gadoxetate disodium-enhanced hepatobiliary phase images. J. Magn. Reson. Imaging.

[17] Bai D.S., Zhang C., Chen P., Jin S.J., Jiang G.Q. (2017). The prognostic correlation of AFP level at diagnosis with pathological grade, progression, and survival of patients with hepatocellular carcinoma. Sci. Rep..

[18] Edoo M.I.A., Chutturghoon V.K., Wusu-Ansah G.K., Zhu H., Zhen T.Y., Xie H.Y., Zheng S.-S. (2019). Serum Biomarkers AFP, CEA and CA19-9 Combined Detection for Early Diagnosis of Hepatocellular Carcinoma. Iran. J. Public Health.

[19] Oka H., Saito A., Ito K., Kumada T., Satomura S., Kasugai H., Osaki Y., Seki T., Kudo M., Tanaka M. (2001). Multicenter prospective analysis of newly diagnosed hepatocellular carcinoma with respect to the percentage of Lens culinaris agglutinin-reactive alpha-fetoprotein. J. Gastroenterol. Hepatol..

[20] Yoshida S., Kurokohchi K., Arima K., Masaki T., Hosomi N., Funaki T., Murota M., Kita Y., Watanabe S., Kuriyama S. (2002). Clinical significance of Lens culinaris agglutinin-reactive fraction of serum alpha-fetoprotein in patients with hepatocellular carcinoma. Int. J. Oncol..

[21] Yan Y., Si Z., Chun C., Chao-Qun P., Ke M., Dong Z., Li W. (2024). Multiphase MRI-Based Radiomics for Predicting Histological Grade of Hepatocellular Carcinoma. J. Magn. Reson. Imaging.

[22] Mao Y., Wang J., Zhu Y., Chen J., Mao L., Kong W., Qiu Y., Wu X., Guan Y., He J. (2022). Gd-EOB-DTPA-enhanced MRI radiomic features for predicting histological grade of hepatocellular carcinoma. Hepatobiliary Surg. Nutr..

[23] He H., Li X., Liu J., Tong Q., Ling M., Zeng Z., Zhou Z. (2024). The Combination of Gd-EOB-DTPA Enhanced T1 Mapping with Apparent Diffusion Coefficient could Improve the Diagnostic Efficacy of Hepatocellular Carcinoma Grading. Curr. Med. Imaging.

[24] Zhao H., Chen C., Gu S., Yan X., Jia W., Mao L., Qiu Y. (2017). Anatomical versus non-anatomical resection for solitary hepatocellular carcinoma without macroscopic vascular invasion: A propensity score matching analysis. J. Gastroenterol. Hepatol..

[25] Qin S., Chen M., O Kaseb A., Kudo M., Lee H.C., Yopp A.C., Zhou J., Wang L., Wen X., Heo J. (2023). Atezolizumab plus bevacizumab versus active surveillance in patients with resected or ablated high-risk hepatocellular carcinoma (IMbrave050): A randomised, open-label, multicentre, phase 3 trial. Lancet.

